# Supplementary material for: The postcranial anatomy of Moschorhinus kitchingi (Therapsida: Therocephalia) from the Karoo Basin of South Africa
Source: PeerJ. 2024 Aug 12;12:e17765. doi: 10.7717/peerj.17765 (PMC11326434; doi:10.7717/peerj.17765)
Supplement: Supplemental Information 1 [file peerj-12-17765-s001.docx]

***Moschorhinus kitchingi* body mass estimation**

We took circumference measurements of the right humerus and the left femur. A small portion of the ventral surface of the shaft of the right humerus is embedded in matrix, thus the complete circumference could not be obtained. However, as the section that is covered is small (<5 mm) we estimate that the complete circumference with the measurable surface (65.45 mm) would be approximately 70.45 mm. The left femur is incomplete, measuring approximately 62% of the length of the complete right femur. The left femur has a clean cross section break allowing for an accurate circumference measurement of 85.68 mm.

Humerus circumference: 70.45 mm

Femur circumference: 85.68 mm

The general equation for terrestrial quadrupeds from Campione and Evans (2012) is:

logBM = 2.749logC_H+F_ - 1.104

where H = humeral circumference and F = femoral circumference

BM = 10^(2.749 * log10(156.13) - 1.104)^

BM = 84 312,89/1000

BM = 84,31 kg
